# Analysis of CT imaging changes of psoas major muscles in patients with lumbar disc herniation mainly based on low back pain and lower limb pain

**DOI:** 10.3389/fsurg.2022.1022903

**Published:** 2023-01-23

**Authors:** Mingchao Cui, Qianru Zhang, Xipeng Chen, Han Wu

**Affiliations:** ^1^ Department of Orthopedics, China-Japan Union Hospital of Jilin University, Changchun, China; ^2^ Department of Orthopedics, The Second Hospital of Tangshan, Tangshan, China; ^3^ Department of Cardiology, Shanghai Ninth People’s Hospital, Shanghai Jiaotong University School of Medicine, Shanghai, China

**Keywords:** lumbar disc herniation, low back pain, lower limb pain, psoas major muscle, atrophy

## Abstract

**Background:**

The study aimed to compare the area changes of CT (computed tomograghy) imaging of psoas major muscle (PM) in patients with lumbar disc herniation (LDH) mainly based on low back pain (LBP) and lower limb pain (LLP), and to analyze the correlation among them.

**Methods:**

We retrospectively analyzed the lumbar CT imaging data of 120 patients with LDH and 60 healthy control people in our hospital from July 2017 to August 2019. They were divided into LBP group (60 cases), LLP group (60 cases) and healthy controls group (60 cases). According to the pain duration and pain degree, LBP group and LLP group were divided into three subgroups respectively. The maximum cross-sectional area (CSA) of PM and the CSA of L5 vertebral body were calculated by Image J software, and the ratio of them was the maximum CSA index of PM. The maximum CSA indices of PM among three groups and three subgroups were compared, respectively.

**Results:**

The baseline data among the three groups weren’t significantly different (*P* > 0.05), yet the maximum CSA index of PM did (*P* < 0.05). In the LBP group, the maximum CSA indices of PM among the three subgroups (short, medium and long) according to the pain duration were significantly different (*P* < 0.05), and those among the three subgroups (light, medium and heavy) according to pain degree did (*P* < 0.05). In the LLP group, the maximum CSA indices of PM among the three subgroups (short, medium and long) were compared, but there was not statistical difference among the three subgroups (*P* > 0.05). No statistical difference in terms of the maximum CSA indices of PM among the three subgroups (light, medium and heavy) was observed (*P* > 0.05).

**Conclusion:**

The atrophy and thinning of PM may be related to LDH. The correlation between the atrophy of PM and LBP was greater than that of LLP. The atrophy of PM in LDH patients with LBP increased with the prolongation of pain duration and aggravation of pain degree.

## Introduction

Lumbar disc herniation (LDH) is a degenerative disease of spine in which the herniated nucleus pulposus compresses or stimulates the sinus nerve and nerve roots, causing clinical symptoms such as low back pain (LBP), lower limb pain (LLP) with or without numbness. The incidence of LDH is higher in middle-aged and elderly patients (1). It was reported that 80% of adults had experienced LBP in their lifetime, and 10% of them had chronic LBP (2), which originated from degenerative lesions of lumbar intervertebral disc that may lead to intervertebral disc herniation. Most of LDH have LBP as the first symptom, with or without limited lumbar movement, and some have some degree of unilateral or bilateral lower limbs numbness and pain. Patients with LBP and radicular pain or radiculopathy have been reported to be more serious and have a worse prognosis than those with only LBP (3). Lumbar computed tomography (CT) or magnetic resonance imaging (MRI) can clearly show the location and degree of nucleus pulposus herniation, which can be used as the basis for the diagnosis of LDH (4, 5).

LDH is caused by some risk factors, such as gender, weight, occupation, and bad living habits, and intervertebral disc degeneration is the main cause (1). However, some studies of spinal biomechanics demonstrated that the main factor of lumbar disc degeneration and even LDH was lumbar imbalance. Psoas major muscle (PM) has been widely studied in terms of lumbar imbalance. It is generally believed that the vertical alignment of PM provides stability for the lumbar spine. However, bilateral asymmetric or atrophic PM can lead to the weakening or asymmetric force of the spinal stability system, which increases the shear stress of the intervertebral disc and causes intervertebral disc herniation. Danneels et al. (6) proposed that the LBP of LDH reduced the activity of PM on the herniated side, leading to its atrophy and thinning. However, the atrophy and thinning of the PM may further aggravate the occurrence and development of LDH. It was thought that only patients with LBP had atrophy and thinning of PM. However, some studies reported that the ipsilateral PM also had atrophy and thinning in cases of LLP caused by LDH (7–9). Kim et al. (10) compared the cross-sectional area (CSA) of the PM on the lesioned and normal side in 76 patients with LDH, but found no statistically significant results.

Currently, the correlation between LDH and atrophy of the PM is highly controversial. Therefore, this study analyzed CT imaging of the PM in LDH patients with mainly based on LBP and LLP and healthy control people, so as to investigate the relationship between LDH and PM, and to analyze its possible mechanism.

## Materials and methods

### Study subjects

From July 2017 to August 2019, the case data of patients with LDH and healthy control people without LDH who underwent lumbar CT scanning in our hospital were retrospectively analyzed. And the study obtained the support of the Ethics Committee of our institution and informed consent of all patients, and was also in accordance with the Helsinki declaration. A total of 180 cases were included in this study and were divided into the LDH with LBP group (LBP group, 60 cases), the LDH with LLP group (LLP group, 60 cases), and the healthy control without LDH group (control group, 60 cases). The inclusion criteria for LBP and LLP groups were as follows: (1) either LBP or LLP, and the pain duration for at least 6 months; (2) unilateral LDH at the L4–L5 level diagnosed by CT or MRI; (3) female 45–65 years old; (4) without a history of heavy physical labor or strengthening training of lumbar muscles. The exclusion criteria for LBP and LLP groups were as follows: (1) patients without LDH; (2) patients with BMI less than 18 or greater than 24; (3) combination of other spinal diseases, such as spinal spondylolisthesis, scoliosis deformity, stenosis, tumors, vertebral fractures, and infections; (4) those with lumbar spine surgery; (5) incomplete imaging data or poor image quality. The inclusion criteria for control group included healthy population without LDH confirmed by lumbar CT or MRI at the same time, women aged 45–65 years old, and no symptoms of LBP or LLP. The exclusion criteria for control group included patients with BMI less than 18 or greater than 24, combined with diseases related to muscle area changes, such as malignant tumors, and incomplete imaging data. Note that LLP is defined as pain or numbness in the back of thigh or down referred to posterior aspect of leg and foot and diagnosed by two independent experienced orthopedic surgeons.

### Procedure

**Figure 1:**
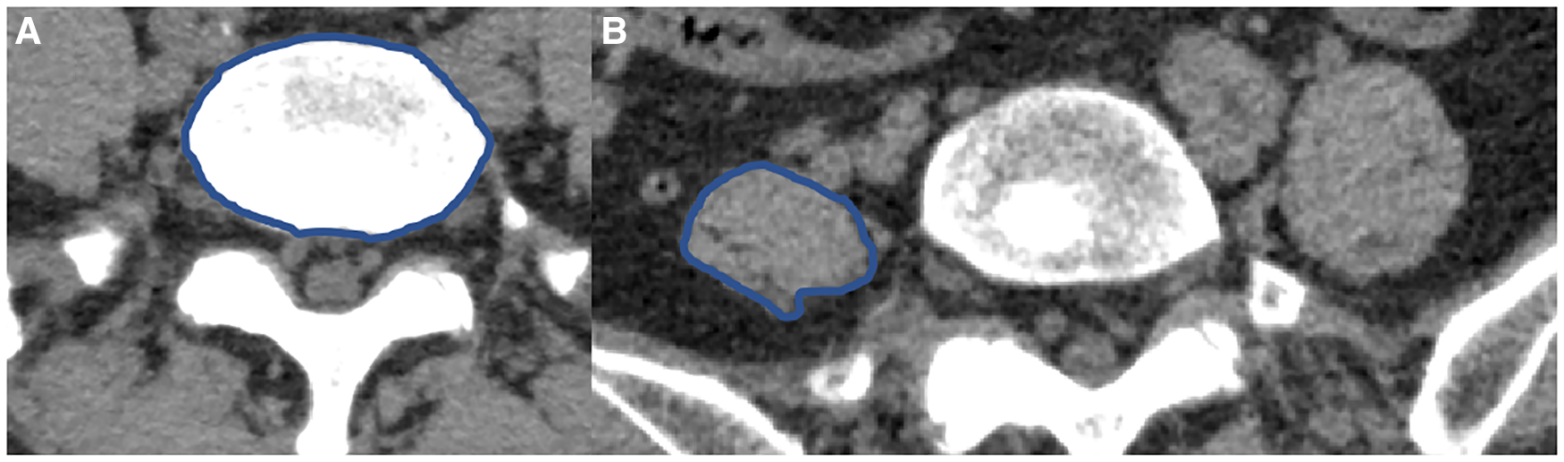
The CSA of L5 vertebral body and PM (**A,B**). (**A**) The CSA of L5 vertebral body. (**B**) The maximum CSA of the left PM.

The lumbar CT images of all participants were reconstructed in three dimensions in our hospital. According to the direction of PM, the maximum horizontal cross section of PM at the side of herniation that was perpendicular to the longitudinal axis of PM, was intercepted on three-dimensional reconstruction CT. The cross section of L5 vertebral body was intercepted at the lower edge of L5 vertebral body (Figure 1A). The maximum CSA of the PM at the side of herniation was measured by Image J soft (National Institutes of Health, USA) (Figure 1B). The ratio of the maximum CSA of the PM divided by the CSA of the L5 vertebral body was used as the maximum CSA index of the PM. The maximum CSA index of the PM was more accurate because it could exclude the interference of some factors, such as height, weight and age (11, 12). All measured data of all participants were recorded.

### Measures

The baseline data, including sex, age and body mass index (BMI), and the visual analog scale (VAS) were collected from medical record. The LBP group and LLP group were divided into 3 subgroups that were short subgroup (6 months ≤ pain duration < 12 months), medium subgroup (12 months ≤ pain duration < 18 months), and long subgroup (18 months ≤ pain duration) on the basis of pain duration, and were also divided into 3 subgroups that were light subgroup (1–3 points), moderate subgroup (4–6 points), and severe subgroup (7–10 points) according to pain degree evaluated by VAS score, respectively. The CSA of L5 vertebral body and the maximum CSA of PM were measured on CT in this study. All the data obtained from the images were measured by two independent professional radiologists.

### Statistical assessments

All the data were enrolled and analyzed by the SPSS 25.0 statistical software (IBM Corporation, USA). The measurement data were expressed as the mean ± standard deviation (M ± SD). The baseline data and the maximum CSA index of the PM among the three groups were compared by one-way analysis of variance (ANOVA), while multiple comparison between groups were compared by Dunnett *t* test. The data that were not normally distributed were compared by nonparametric test. The count data were compared by chi-square test. The difference was statistically significant if *P* < 0.05.

## Results

No statistical difference was found among the three groups in terms of the mean age and BMI (*P* = 0.129 and *P* = 0.771, respectively) (Table 1). There was statistical difference among the three groups with regards to the maximum CSA index of PM (0.55 ± 0.09 vs. 0.61 ± 0.07 vs. 0.71 ± 0.08, *F* = 60.66, *P* < 0.001) (Table 2). In the LBP group, the maximum CSA indices of PM among the three subgroups (short, medium and long) according to the pain duration were significantly different (0.66 ± 0.04 vs. 0.55 ± 0.03 vs. 0.45 ± 0.04, *F* = 135.48, *P* < 0.001), and those among the three subgroups (light, medium and heavy) according to pain degree did (0.66 ± 0.04 vs. 0.53 ± 0.05 vs. 0.42 ± 0.04, *F* = 90.79, *P* < 0.001) (Table 3). In the LLP group, the maximum CSA indices of PM among the three subgroups (short, medium and long) were compared, but there was not statistical difference among the three subgroups (0.64 ± 0.07 vs. 0.61 ± 0.07 vs. 0.58 ± 0.07, *P* = 0.085). No statistical difference in terms of the maximum CSA indices of PM among the three subgroups (light, medium and heavy) was observed (0.64 ± 0.07 vs. 0.60 ± 0.08 vs. 0.58 ± 0.05, *P* = 0.090) (Table 4).

**Table 1:** Comparison of baseline data among the three groups.

Variables	LBP group (*n* = 60)	LLP group (*n* = 60)	Control group (*n* = 60)	*P* value
Age (years)	57.58 ± 5.73	56.28 ± 5.76	55.53 ± 5.25	0.129
BMI (kg/m^2^)	23.03 ± 0.87	23.15 ± 0.87	23.11 ± 0.86	0.771

Values are expressed as the mean ± SD, number (%), or as otherwise indicated. LBP, low back pain; LLP, lower limb pain; BMI, body mass index.

**Table 2:** Comparison of the maximum CSA indices of PM among three groups.

Group	M ± SD	95% CI	*F*-test	
			*F* value	*P* value
LBP group	0.55 ± 0.09	0.53–0.58	60.66	0.000*
LLP group	0.61 ± 0.07	0.59–0.63		
Control group	0.71 ± 0.08	0.69-0.73		

Values are expressed as the mean ± SD, number, or as otherwise indicated. LBP, low back pain; LLP, lower limb pain; CSA, cross-sectional area; PM, psoas major muscle; CI, confidence interval.

* *P* < 0.05 was considered to be statistically significant.

**Table 3:** Comparison of the maximum CSA indices of PM of three subgroups in the LBP group.

Variables	Pain duration			Pain degree		
	Short (*n* = 18)	Medium (*n* = 23)	Long (*n* = 19)	Light (*n* = 18)	Medium (*n* = 34)	Heavy (*n* = 8)
CSA index	0.66 ± 0.04	0.55 ± 0.03	0.45 ± 0.04	0.66 ± 0.04	0.53 ± 0.05	0.42 ± 0.04
*F* value	135.48			90.79		
*P* value	0.000* (short versus medium, *P* = 0.000*; short versus long, *P* = 0.000*)			0.000* (light versus medium, *P* = 0.000*; light versus heavy, *P* = 0.000*)		

Values are expressed as the mean ± SD, number, or as otherwise indicated. LBP, low back pain; PM, psoas major muscle; CSA, cross-sectional area.

*P < 0.05 was considered to be statistically significant.

**Table 4:** Comparison of the maximum CSA indices of PM of three subgroups in the LLP group.

Variables	Pain duration			Pain degree		
	Short (*n* = 17)	Medium (*n* = 26)	Long (*n* = 17)	Light (*n* = 20)	Medium (*n* = 30)	Heavy (*n* = 10)
CSA index	0.64 ± 0.07	0.61 ± 0.07	0.58 ± 0.07	0.64 ± 0.07	0.60 ± 0.08	0.58 ± 0.05
*F* value	2.57			2.52		
*P* value	0.085 (short versus medium, *P* = 0.268; short versus long, *P* = 0.050)			0.090 (light versus medium, *P* = 0.190; light versus heavy, *P* = 0.074)		

Values are expressed as the mean ± SD, number, or as otherwise indicated. LLP, lower limb pain; PM, psoas major muscle; CSA, cross-sectional area.

## Discussion

Previous studies have shown that the atrophic changes of PM are associated with unilateral LBP (13, 14). In addition, PM was smaller in patients with LBP compared to controls (15). In this study, we found that the atrophy and thinning of PM may be associated with LDH. Moreover, the correlation between the atrophy of PM and LBP was greater than that of LLP.

There was statistical difference among the three groups in terms of the maximum CSA index of PM. This showed that LDH was correlated with the atrophy of PM. At present, the mechanism of psoas muscle atrophy caused by LDH is uncertain. In our opinion, LBP or LLP limited the activity of PM, which may be the cause of psoas muscle atrophy. Moreover, the herniated intervertebral disc may limit the activity of the ipsilateral PM by stimulating the ipsilateral PM to produce pain, which may lead to atrophy of the PM. With the increase of age, muscle tissue may atrophy to varying degrees, and the decrease of muscle may reduce the tension and strength of muscle (16, 17). However, the atrophy and thinning of PM reduced the stability of the spine and caused lumbar imbalance due to weaken the lumbar support force or uneven stress, and the asymmetric changes of bilateral PMs increased the shear forces between spinal segments, aggravated the degenerative changes of intervertebral disc and increased the possibility of LDH (10). The interaction between LDH and PM may be a so-called vicious circle. A prospective study conducted by Dangaria demonstrated that the CSA of the ipsilateral PM was reduced in unilateral LDH (7). Our results were the same as those of the prospective study. More importantly, our study also found that LBP had a greater impact on the atrophy of PM compared with LLP. This may be due to the fact that LBP was more restrictive to human activity as well as PM movement compared to LLP.

In LBP groups, the maximum CSA indices of PM between the two subgroups (short, long) according to the pain duration and between the two subgroups (light, heavy) according to the pain degree were significantly different, but there was no statistical difference in LLP groups. This revealed that pain duration and pain degree of LBP were related to the atrophy of PM. The severe the pain degree and the longer the pain duration, the more severe the atrophy of the PM and the smaller area of the PM, which seemed to be related to the lower activity of the patients. A recent study showed that preoperative atrophy of the PM may affect the outcome of surgery for lumbar spine disease (18). Patients with LDH, especially with LBP symptoms, should be treated as soon as possible by appropriate treatment methods to avoid the atrophy of PM. Compared to before exercise, Tetsushi et al. (19) reported that PM may increase significantly in a short period of time after exercise. A study by Tawara et al. (20). demonstrated that the PM increased significantly on an exercised side compared to the non-exercised side. If patients suffer from LDH mainly based on LBP and LLP, especially those with long pain duration and severe pain, they should be treated as early as possible and take appropriate activities and exercises to avoid atrophy of PM.

There were some limitations in the study. Firstly, to some extent, there may be selection bias in this retrospective study. Secondly, the sample size was small in this study, with only 180 patients. Prospective randomized controlled trials with large sample size and multicenter are still needed to better investigate the correlation between LDH and PM, and to analyze its possible mechanism.

## Conclusion

The atrophy and thinning of PM may be associated with LDH. The correlation between the atrophy of PM and LBP was greater than that of LLP. The atrophy of PM in LDH patients with LBP increased with the prolongation of pain duration and aggravation of pain degree.

## Data Availability

The raw data supporting the conclusions of this article will be made available by the authors, without undue reservation.
